# KANK2 Links αVβ5 Focal Adhesions to Microtubules and Regulates Sensitivity to Microtubule Poisons and Cell Migration

**DOI:** 10.3389/fcell.2020.00125

**Published:** 2020-03-03

**Authors:** Mladen Paradžik, Jonathan D. Humphries, Nikolina Stojanović, Davor Nestić, Dragomira Majhen, Ana Dekanić, Ivana Samaržija, Delphine Sedda, Igor Weber, Martin J. Humphries, Andreja Ambriović-Ristov

**Affiliations:** ^1^Laboratory for Cell Biology and Signalling, Division of Molecular Biology, Ruđer Bošković Institute, Zagreb, Croatia; ^2^Wellcome Centre for Cell-Matrix Research, Faculty of Biology, Medicine and Health, University of Manchester, Manchester, United Kingdom; ^3^Laboratory of Cell Biophysics, Division of Molecular Biology, Ruđer Bošković Institute, Zagreb, Croatia

**Keywords:** adhesome, integrin αVβ5, antitumor drug resistance, cell migration, cortical microtubule stabilizing complex, KANK2

## Abstract

Integrins are heterodimeric glycoproteins that bind cells to extracellular matrix. Upon integrin clustering, multimolecular integrin adhesion complexes (IACs) are formed, creating links to the cell cytoskeleton. We have previously observed decreased cell migration and increased sensitivity to microtubule (MT) poisons, paclitaxel and vincristine, in the melanoma cell line MDA-MB-435S upon transfection with integrin αV-specific siRNA, suggesting a link between adhesion and drug sensitivity. To elucidate the underlying mechanism, we determined αV-dependent changes in IAC composition. Using mass spectrometry (MS)-based proteomics, we analyzed the components of isolated IACs of MDA-MB-435S cells and two MDA-MB-435S-derived integrin αV-specific shRNA-expressing cell clones with decreased expression of integrin αV. MS analysis showed that cells preferentially use integrin αVβ5 for the formation of IACs. The differential analysis between MDA-MB-435S cells and clones with decreased expression of integrin αV identified key components of integrin αVβ5 adhesion complexes as talins 1 and 2, α-actinins 1 and 4, filamins A and B, plectin and vinculin. The data also revealed decreased levels of several components of the cortical microtubule stabilization complex, which recruits MTs to adhesion sites (notably liprins α and β, ELKS, LL5β, MACF1, KANK1, and KANK2), following αV knockdown. KANK2 knockdown in MDA-MB-435S cells mimicked the effect of integrin αV knockdown and resulted in increased sensitivity to MT poisons and decreased migration. Taken together, we conclude that KANK2 is a key molecule linking integrin αVβ5 IACs to MTs, and enabling the actin-MT crosstalk that is important for both sensitivity to MT poisons and cell migration.

## Introduction

Integrins are cell-surface adhesion molecules that connect cells to other cells and components of the ECM. Integrins are heterodimers composed of α and β subunits. A set of 18 α and 8 β subunits that associate to form 24 different αβ heterodimers have thus far been identified. Integrins are involved in signaling pathways that regulate many essential cellular functions including survival, proliferation and migration (Desgrosellier and Cheresh, 2010; Cooper and Giancotti, 2019). Upon clustering of integrins, other proteins are recruited to their cytoplasmic tails to form multimolecular IACs which establish the linkage between integrins and the cell cytoskeleton (Winograd-Katz et al., 2014). Integrins together with their associated IAC components have been termed the adhesome (Zaidel-Bar et al., 2007; Kuo et al., 2011; Schiller et al., 2011; Byron et al., 2015; Jones et al., 2015). The fate of such a complex depends on cell type, composition of ECM, matrix stiffness, integrin subtype, etc. ultimately leading to formation of a variety of IAC-induced structures including nascent adhesions, FAs, fibrillar adhesions, podosomes and invadopodia (Klapholz and Brown, 2017; Gough and Goult, 2018). Novel adhesive structures termed reticular adhesions (RAs) were identified in 2018, which are rich in integrin αvβ5 but devoid of most of the typical cytoskeletal components found in other IACs, and, instead, are connected to the clathrin machinery (Lock et al., 2018). The association between integrins and F-actin occurs through proteins such as talin and vinculin (Atherton et al., 2015). Talin also coordinates the microtubule (MT) cytoskeleton at adhesion sites through the interaction with KN motif and ankyrin repeat domain-containing (KANK) proteins (Bouchet et al., 2016; Sun et al., 2016), which stimulates FA turnover (Stehbens and Wittmann, 2012).

Several protocols have been developed to purify ventral IACs from 2D cell cultures (Humphries et al., 2009; Kuo et al., 2011; Schiller et al., 2011; Jones et al., 2015). A variety of cell types have been investigated using these methods. However, most IAC preparations have been isolated from cells seeded on fibronectin. Seven of these datasets have been analyzed together which led to the definition of a fibronectin-induced “meta adhesome” composed of over 2400 proteins. The meta adhesome was further reduced to 60 core proteins, termed as the “consensus adhesome” i.e., proteins that are most frequently identified in IACs (Winograd-Katz et al., 2014; Horton et al., 2015). The consensus adhesome was obtained from cells seeded on fibronectin, and therefore its composition reflects components of integrin heterodimers α5β1 and αVβ3 (Horton et al., 2016). It was also shown to be centered around potential axes that link integrins to actin, namely ILK-PINCH-parvin-kindlin, FAK-paxillin, talin-vinculin and α-actinin-zyxin-VASP axis (Humphries et al., 2019).

The repertoire of integrins in tumor cells and the composition of ECM, deposited by tumor and stromal cells, both contribute to integrin signaling in promoting invasive growth and metastasis (Cooper and Giancotti, 2019). Integrin signaling confers resistance of cancer cells to chemotherapy (Stojanovic et al., 2016) and radiotherapy (Eke et al., 2018). Conversely, knockdown of integrins may sensitize tumor cells to both chemo- and radiotherapy (Stojanovic et al., 2018; Wang et al., 2019). Therefore, integrins are promising targets to be combined with classical therapy, especially αV integrins which play non-essential roles in development but are involved in tumor growth and angiogenesis (Seguin et al., 2015). The integrin αV subunit is known to form heterodimers with one of five different β subunits (β1, β3, β5, β6, and β8) (Hynes, 2002). Among these integrins, αVβ3 and αVβ5 have been extensively studied. Integrin αVβ3, highly expressed in melanoma (Nip et al., 1992; Danen et al., 1995), has a pivotal role in melanoma growth (Mitjans et al., 2000), initiates the transition from the benign radial growth phase to the malignant vertical growth phase (Albelda et al., 1990), and its expression is increased in brain metastases compared to primary melanoma (Vogetseder et al., 2013). The importance of integrin αVβ5 was also demonstrated in melanoma, showing its involvement in the highly aggressive phenotype of melanoma cells expressing neuropilin 1 (Ruffini et al., 2015). Integrin αVβ5 has been shown to trigger formation of FAs (De Deyne et al., 1998). Moreover, it has been recently shown that integrin αVβ5 is the predominant integrin used by cells in long term culture, not only in FAs but also in RAs. RAs are morphologically and dynamically distinct from classical FAs, formed during interphase and preserved at cell-ECM attachment sites throughout cell division (Lock et al., 2018, 2019).

Using the melanoma cell line MDA-MB-435S, which expresses only integrins β3 and β5 as αV integrins binding partners, we showed that αV knockdown increases sensitivity to antitumor drugs that target MTs, paclitaxel (PTX) and vincristine (VCR). We also identified integrin αVβ5 as a key integrin in this process. Concomitantly, we demonstrated that integrin αV knockdown decreases migration and invasion, a process in which both integrins αVβ3 and αVβ5 are involved (Stojanovic et al., 2018). Here, we aimed to identify the integrin(s) used by MDA-MB-435S cells in cell culture and to catalog changes in IAC composition upon integrin αV knockdown. We report that integrin αVβ5 is the predominant integrin used by MDA-MB-435S cells in long term culture and reveal the composition of integrin αVβ5 adhesion complexes. We show that one of the identified components is KANK2, whose knockdown in MDA-MB-435S cells mimics increased sensitivity to MT poisons and decreased migration upon integrin αV knockdown. Therefore, KANK2 is crucial for the functional connection of integrin αVβ5-containing FAs with MTs and represents a potential target for improvement of melanoma therapy.

## Materials and Methods

### Cells and Isolation of Stable Cell Clones

Human melanoma cell line MDA-MB-435S (a spindle-shaped variant of the parental MDA-MB-435) was obtained from the American Type Culture Collection (ATCC, United States). Cells were grown in DMEM (Invitrogen, United States), and supplemented with 10% (v/v) FBS (Invitrogen, United States) at 37°C with 5% CO_2_ (v/v) in a humidified atmosphere. To construct MDA-MB-435S clones with decreased expression of integrin αV subunit, pSUPER vector system for the expression of shRNA was used (OligoEngine, United States). Briefly, oligonucleotides were constructed according to the sequence of siRNA specific for integrin subunit αV (s7568- Ambion, United States), previously used in transient transfection in the same cell model (Stojanovic et al., 2018). Oligonucleotides P1 – GATCCCCGAATATCGGTTGGATTATATTCAAGAGA TATAATCCAACCGATATTCTTTTTA and P2 – AGCTTAAA AAGAATATCGGTTGGATTATATCTCTTGAATATAATCCAA CCGATATTCGGG were annealed and cloned into *Hin*dIII (NEB, United Kingdom) and *Bgl*II (Thermo Fisher Scientific, United States) digested vector. Resulting plasmid pSuper shαV was purified using Miniprep columns (Qiagen, Germany) and transfected into MDA-MB-435S cells using Lipofectamine (Thermo Fisher Scientific, United States). Clones 2αV and 3αV were selected using puromycin (0.2 μg/ml, Sigma-Aldrich, United States) according to expression of integrin αV measured by flow cytometry.

### Drugs, Chemicals, Determination of Cell Survival, and Transient siRNA Transfection

*Cis*-diamminedichloroplatinum (cisplatin, CDDP) was dissolved in water and stored at −20°C. Vincristine and paclitaxel (all Sigma-Aldrich, United States) were dissolved in phosphate-buffered saline (PBS) and DMSO, respectively, and stored at −20°C. The 3-((4,5-dimethylthiazol-2-yl)-2,5-diphenyltetrazolium bromide) (MTT) (Millipore, United States) was dissolved in PBS and stored at 4°C. The sensitivity of cells to antitumor drugs was determined using MTT assay. Briefly, 24 h after seeding in 96-well tissue culture plates (1–1.2 × 10^4^ cells/well) cells were treated with different concentrations of antitumor drugs. Seventy-two hours later, the absorbance of MTT-formazan product dissolved in DMSO was measured with a microplate reader (Awareness Technology, Inc., United States) at 600 nm. Single siRNA specific for KANK2 (target sequence ATGTCAACGTGCAAGATGA; Sigma-Aldrich) was used at 20 nM, validated by western blot analysis (WB) and immunofluorescence (IF) using KANK2 specific antibody as previously described (Bouchet et al., 2016) (Supplementary Table S1). Twenty-four hours after KANK2 knockdown cells were used in MTT or migration assays.

### Determination of Cell Migration

For monitoring cell migration, serum-starved (24 h) cells [8 × 10^4^ cells in 0.5 mL of DMEM containing 0.1% (w/v) BSA] were placed in the Transwell Cell Culture Inserts (pore size, 8 μm) (Corning, United States) and left to migrate for 22 h toward 10% (v/v) FBS in DMEM as a chemoattractant. Cells on the upper side of the filters were removed with cotton-tipped swabs, the filters were fixed in paraformaldehyde for 15 min and stained with 1% (w/v) crystal violet in PBS for 90 min. Cells on the underside of the filters were photographed using Olympus BX51TF microscope (five images/sample). The number of cells was determined using ImageJ (NIH, United States) software.

### Determination of Integrin αVβ3, αVβ5, αV, and β1 Expression by Flow Cytometry

Flow cytometry to analyze the expression of αVβ3, αVβ5, αV, and β1 was performed using integrin subunit- or heterodimer-specific monoclonal antibodies (MAb). Briefly, adherent cells were grown in tissue culture dishes, detached by EDTA (Invitrogen, United States) and washed twice with PBS. Membrane fluorescence staining was performed using unlabeled primary antibodies (1 h, 4°C) while its binding was revealed by incubation (30 min, 4°C) of FITC-conjugated anti-mouse antibody. Isotype control samples were incubated with mouse IgG1 followed by FITC-conjugated anti-mouse antibody. Flow cytometry experiments were performed using FACSCalibur, while cell acquisition was made using BD CellQuest software package (all BD Biosciences, United States). Data were analyzed using FCS Express 3 (*De Novo* Software, United States) software. All antibodies are listed in Supplementary Table S1.

### Assessment of Apoptosis and Cell Proliferation

The induction of apoptosis in MDA-MB-435S, 2αV, and 3αV cells was determined by the Annexin V-FITC (BD Pharmingen, Germany)/PI double-staining. Cells were treated for 72 h with PTX (0.004 μg/mL) and apoptosis was measured by flow cytometry. To monitor cell proliferation, Click-iT^®^ assay was used according to the manufacturer’s instructions (Thermo Fisher Scientific, United States). Briefly, 2.75 × 10^5^ cells/well were seeded on 6-well plate and grown for 72 h in DMEM supplemented with 10% (v/v) FBS. Two hours before harvesting, modified thymidine analog EdU (5-ethynyl-2′-deoxyuridine, final concentration 10 μM) was added. Cells were collected, fixed with 4% (w/v) paraformaldehyde, permeabilized with saponin, stained with AF 488 azide (in the presence of CuSO_4_) and analyzed by flow cytometry. To determine the proliferation rate, the frequencies of the proliferative (EdU +) cells were compared.

### Confocal Microscopy and Live Cell Imaging

For confocal microscopy, 48 h after being seeded on coverslips, cells were fixed with 2% (w/v) paraformaldehyde (methanol was used only when staining of α-tubulin/KANK2 was performed), permeabilized with 0.1% (v/v) Triton X-100, incubated with the appropriate antibodies for 1 h, followed by incubation with the appropriate secondary antibody for 1 h. F-actin fibers were stained with rhodamine phalloidin (Sigma-Aldrich, United States) while MTs were stained with antibody against α-tubulin (Sigma-Aldrich, United States), and slides mounted in DAPI Fluoromount-G (SouthernBiotech, United States) (all antibodies are listed in Supplementary Table S1). Fluorescence and respective IRM images were acquired using HC PL APOCS2 63 × /1.40 oil-immersion objective on an inverted confocal microscope (Leica TCS SP8 X, Leica Microsystems, Germany), with the focus adjusted to the adhesion sites of cells at the upper surface of glass coverslip (Weber, 2003). Images were analyzed using LAS X (Leica Microsystems, Germany) and ImageJ (NIH, United States) software. For quantification of FA proteins/KANK2, images were processed using ImageJ and threshold was set to restrict analysis to sites where the signals from the protein staining overlaps with the F-actin/MT staining at the tip of the actin stress fibers/MT fibers. For the stress fiber quantification, only those fibers that end with FAs, marked by paxillin staining, were identified as stress fibers and quantified using ImageJ. For time-lapse live cell imaging, cells were seeded on 35 mm glass bottom dishes (Ibidi, Martinsried, Germany) and 2–3 fields containing cells were imaged every 44 s for 18–20 h using HC PL APOCS2 40 × /1.30 oil-immersion objective on the Leica TCS SP8 X microscope equipped with a top stage incubator at 37°C. Images were analyzed using LAS X. EVOS cell imaging system (Thermo Fisher Scientific, United States) was used to obtain cell morphology images of cells seeded in 6-well plates, every 24 h for a 72 h period. Images were analyzed using ImageJ (NIH, United States) software.

### Isolation of IACs, Sample Preparation for Mass Spectrometry, and Data Analysis

Integrin adhesion complexes were isolated as previously described (Jones et al., 2015). In short, cells (2–2.5 × 10^6^, depending on cell clone, to obtain similar cell number 48 h later) were plated on 10 cm diameter cell culture dishes (at least six dishes per cell line) and grown in DMEM supplemented with 10% (v/v) FBS. After 48 h, the medium was removed, cells were washed with DMEM-HEPES and incubated with Wang and Richard’s reagent for 5 min (6 mM DTBP, Thermo Fisher Scientific, United States). At the same time, cells in an additional plate were counted to ensure equal cell number per sample. DTBP was quenched by 0.03M Tris-HCl (pH 8) and cells were lysed using modified RIPA buffer. Cell bodies were removed by high-pressure washing and remaining adhesions were collected by scraping. Samples containing isolated IACs were acetone precipitated, dissolved in Laemmli buffer and further processed either for MS or WB analysis.

Samples were prepared as previously described (Humphries et al., 2009), using a slightly modified procedure. Briefly, samples were loaded onto gradient gels (NuPage 4–12% Bis-Tris protein gels, Thermo Fisher Scientific, United Kingdom) and electrophoresis was performed for 3 min (200V). Protein bands were stained with InstantBlue (Expedeon, United Kingdom), followed by excision and destaining using series of alternate washing steps with ammonium bicarbonate (Fluka, United States) and acetonitrile (Thermo Fisher Scientific, United Kingdom). Washing and drying steps were made in 96-well perforated plates (GlySci, United States). Gel pieces were dried with acetonitrile and additional drying was performed using vacuum centrifuge. Samples were reduced with dithiothreitol (DTT, 1 h at 56°C) and alkylated using 55 mM iodoacetamide (37°C, 45 min, dark) (both Sigma-Aldrich, United States). After series of washing and drying steps, gel pieces were incubated with trypsin (1.25 ng/μL, Promega, United States) and incubated for 45 min at 4°C, followed by an overnight incubation at 37°C. Peptides were collected and extracted using acetonitrile supplemented with formic acid (Sigma-Aldrich, United States), then dried and resuspended in a solution of 5% (v/v) acetonitrile plus 0.1% (v/v) formic acid. Peptides were desalted using OLIGO R3 beads (Life technologies, United States) using 96-well plates with PVDF membranes (Corning, United States). Desalting was performed with a 0.1% formic acid wash steps before being eluted twice with 50% acetonitrile in 0.1% formic acid. Peptides were subsequently dried and resuspended in a solution of 5% (v/v) acetonitrile plus 0.1% (v/v) formic acid for LC-MS/MS analysis.

Samples were analyzed using a modified version of the LC-MS/MS method previously described (Horton et al., 2015). Briefly, an UltiMate^®^ 3000 Rapid Separation LC (RSLC, United States) coupled to an Orbitrap Elite mass detector (Thermo Fisher Scientific, United States) with electrospray ionization. Peptide mixtures were eluted for 44 min using a gradient containing 92% of solution A (0.1% formic acid in water) and 8% up to 33% of solution B (0.1% formic acid in acetonitrile). Solvent flow was set to 300 nL per minute. To identify proteins, data were searched against the human Swissprot database (version 2018_01) using Mascot (Matrix science, version 2.5.1). Fragment ion tolerance was set to 0.50 Da, while parent ion tolerance was 5 PPM. Protein identifications were further refined using Scaffold (Proteome software). Protein (99.9%) and peptide (95%) probabilities were assigned using the Protein Prophet algorithm (Nesvizhskii et al., 2003) as incorporated by Scaffold including a minimum of four unique peptides per each protein.

### PPI Network Formation, Functional Enrichment, Gene Ontology Analysis, and MS Data Visualization

Human protein–protein interactions were loaded from STRING database, using stringApp (confidence score cut-off = 0.40, maximum additional interactors = 0) (Doncheva et al., 2019) for Cytoscape software (version 3.7.1) (Shannon et al., 2003). Functional annotation was performed using the Database for annotation, visualization and integrated discovery (DAVID), version 6.8 (Huang da et al., 2009a) and Panther GO database (Thomas et al., 2003), while the literature search was performed in case of cortical stabilization microtubule complex (Lansbergen et al., 2006; Astro et al., 2014; Bouchet et al., 2016; Noordstra and Akhmanova, 2017).

Functional enrichment was performed using DAVID_CC subontology list (Benjamini–Hochberg corrected *P*-value < 0.05, EASE score < 0.1, at least four identified proteins). To summarize the gene ontology terms and place them in similarity-based space, REViGO tool, with the following setup (comparison of corrected *P*-values related to GO terms were used, allowed similarity: small (0.5), semantic similarity measure to use: Resnik-normalized) was used (Supek et al., 2011). QSpec Spectral counter tool was used to provide the statistical measure of differentially expressed proteins in MDA-MB-435S versus 2αV datasets and MDA-MB-435S versus 3αV datasets (Choi et al., 2008). For visualization of differentially expressed proteins, volcano plot (GraphPad Prism) with the following setup was created: fold change > 1.5 (MDA MB 435S/2αV and MDA MB 435S/3αV, respectively), −log(FDR) > 1. Fold change was calculated according to Qspec output values.

### Western Blot Analysis

Isolated IACs from at least six 10 cm diameter culture dishes were mixed with 2x loading buffer and heated for 20 min at 70°C. Proteins were loaded onto gradient pre-cast gels (Biorad, United States), separated with SDS-PAGE and transferred to a nitrocellulose membrane (Amersham, Germany). For assessment of successful KANK2 knockdown cells were lysed using RIPA buffer, mixed with 5x loading buffer, separated by SDS-PAGE and transferred to nitrocellulose membrane (Amersham, Germany). The membrane was blocked in 5% (w/v) non-fat dry milk, and incubated with the appropriate antibodies, followed by incubation with horseradish peroxidase-coupled secondary antibody (GE Healthcare, United States, Invitrogen, United States) (Supplementary Table S1). Detection was performed using chemiluminescence (GE Healthcare) and visualized using iBright CL1000 (Thermo Fisher Scientific, United States).

### Statistical Analysis

Each experiment was repeated at least three times, and GraphPad Prism v5.0 (GraphPad Software, United States) was used to analyze the data. All data from MTT experiments were analyzed by unpaired Student’s *t*-test, and expressed as mean ± standard deviation (SD). ns, not significant; ^∗^*P* < 0.05; ^∗∗^*P* < 0.01; ^∗∗∗^*P* < 0.001. Data obtained from migration and IF were analyzed by related measure one-way ANOVA with Dunnett’s multiple comparison: ^∗^*P* < 0.05, ^∗∗^*P* < 0.01, ^∗∗∗^*P* < 0.001.

## Results

### MDA-MB-435S Clones With Stable Knockdown of Integrin αV Subunit Display Increased Sensitivity to Microtubule Poisons and Decreased Cell Migration

**FIGURE 1:**
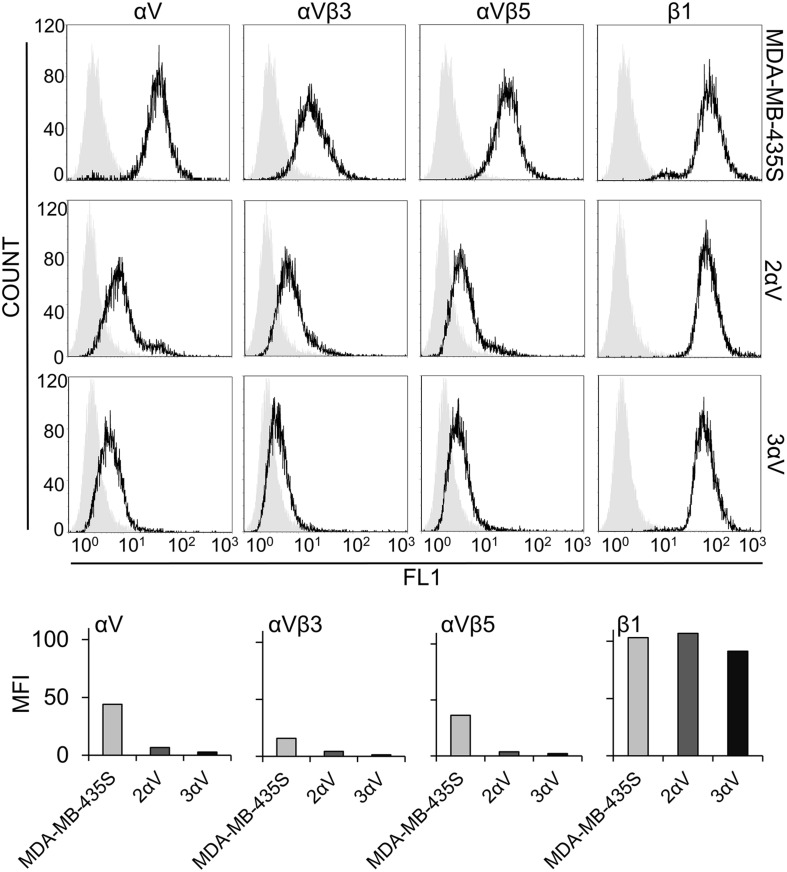
Characterization of the MDA-MB-435S cell model. Surface expression of integrin subunit αV, integrin heterodimers αVβ3 and αVβ5, and integrin subunit β1 in melanoma cell line MDA-MB-435S and clones 2αV and 3αV obtained by stable transfection with integrin αV-specific shRNA. Cells were detached by EDTA and analyzed by flow cytometry upon incubation with antibodies against integrin subunit αV, β1, or integrin heterodimers αVβ3 or αVβ5 (black histogram), and isotype-matched antibody as a negative control (gray histogram), followed by rabbit FITC-conjugated-anti-mouse antibody (upper panel). Results from upper panel were presented as comparisons of MFIs within the cell model (lower panel). Representative data of three independent experiments yielding similar results are shown.

**FIGURE 2:**
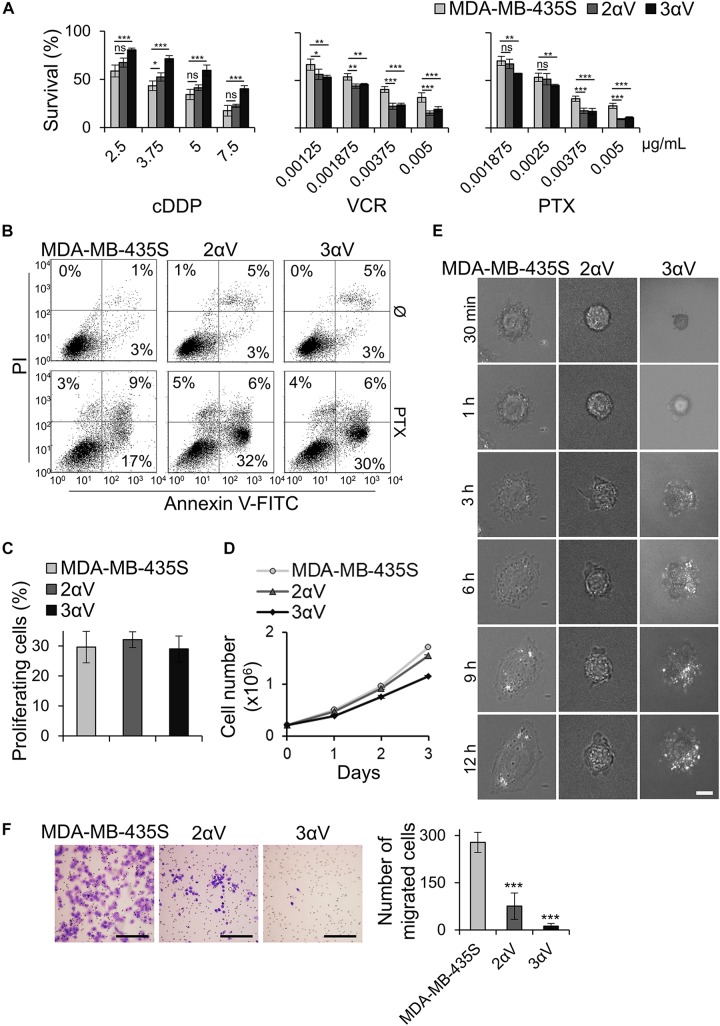
Effect of integrin αV knockdown on cell sensitivity to antitumor drugs, PTX-induced apoptosis, cell proliferation, growth, spreading and migration. **(A)** Clones 2αV and 3αV demonstrate decreased sensitivity to cDDP and increased sensitivity to VCR and PTX as compared to parental MDA-MB-435S cells. Cells were seeded in 96-well plates and 24 h later treated with different concentrations of cDDP, VCR, and PTX. Cytotoxicity was measured by MTT assay. Results presented are representative of three independent experiments with similar results ± SD. Data were analyzed by unpaired Student’s *t*-test. ns, not significant; *P < 0.05; **P < 0.01; ***P < 0.001. **(B)** Clones 2αV and 3αV upon PTX treatment demonstrate increased apoptosis as compared to MDA-MB-435S cells. Cells were treated with 0.004 μg/mL of PTX for 72 h, harvested for Annexin V/PI staining and analyzed by flow cytometry to discriminate between live (lower left quadrant), apoptotic and/or necrotic cells (right quadrants). The representative data of three independent experiments yielding similar results are shown. **(C)** Cell proliferation in MDA-MB-435S cells and clones 2αV and 3αV. Cell proliferation was measured using ClickIT EdU assay. DNA synthesis was measured upon 2 h cell growth in medium supplemented with EdU and the amount of incorporated EdU was measured by flow cytometry. Comparison of average percentage of EdU + cells from three different experiments are shown. **(D)** Growth curve of MDA-MB-435S cells and clones 2αV and 3αV. Cells were seeded in 10 cm culture plates and counted on days 1, 2, and 3. The results presented are representative of three independent experiments with similar results. **(E)** Cell spreading of MDA-MB-435S cells and clones 2αV and 3αV. Live cell imaging was performed during 18–20 h upon seeding and cell spreading was compared using time lapse IRM images (30 min–12 h). Scale bar = 10 μm. **(F)** Decreased migration of clones 2αV and 3αV as compared to MDA-MB-435S cells. Serum starved (24 h) cells were seeded in Transwell cell culture inserts and left to migrate for 22 h toward serum. Cells on the underside of the inserts were stained with crystal violet, photographed, and counted. Scale bar = 100 μm. Averages of five microscope fields of three independently performed experiments ± SD are shown (*n* = 3). Data were analyzed by one-way ANOVA with Dunnett’s multiple Comparison. ***P < 0.001.

**FIGURE 3:**
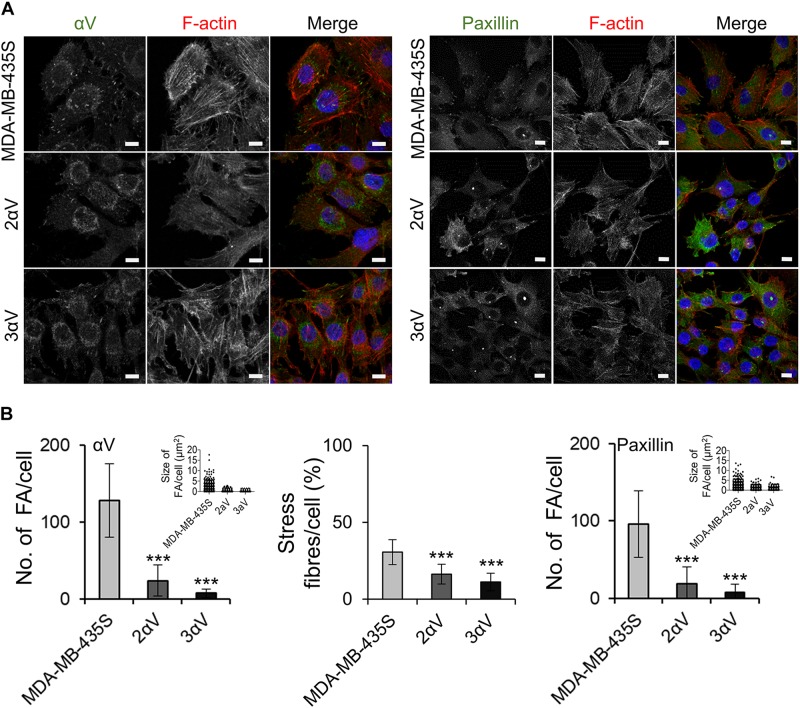
Clones 2αV and 3αV have decreased number and size of FAs and decreased amount of stress fibers per cell as compared to MDA-MB-435S cells. **(A)** Clones 2αV and 3αV show decreased expression of the integrin subunit αV and the FA marker, paxillin. Forty-eight hours after seeding on coverslips, cells were fixed, permeabilized and stained with antibody against integrin αV or paxillin followed by Alexa-Fluor 488-conjugated antibody (green). F-actin staining (red) was performed in all samples, and nuclei were stained with DAPI (blue). Analysis was performed using TCS SP8 Leica. Scale bar = 10 μm. **(B)** Quantification of αV and paxillin, FA size and % of stress fibers per cell. Data presented as histograms or scatter plots (FA size) represent measurements of > 50 cells and are plotted as mean ± SD (*n* = 3). Data were analyzed by one-way ANOVA with Dunnett’s multiple comparison. ***P < 0.001.

We have recently shown that knockdown of integrin subunit αV, via transient transfection of integrin αV-specific siRNA, increased sensitivity of MDA-MB-435S cells to PTX and VCR and decreased migration. In a series of experiments, the effect on sensitivity to MT poisons was ascribed to integrin αVβ5 but not to integrin αVβ3. On the other hand, both αV integrins, αVβ3 and αVβ5, were implicated in the regulation of cell migration (Stojanovic et al., 2018). In the present work, our aim was to unravel a possible link between these alterations in sensitivity to MT poisons and migration and the adhesome composition. When β5 was knocked down, we observed upregulation of integrin αVβ3, and vice versa. We named this phenomenon the integrin balance effect (Stojanovic et al., 2018). For this reason, we cataloged the adhesome of MDA-MB-435S cells and compared it to the adhesome of cells upon integrin αV knockdown. We isolated two MDA-MB-435S clones stably transfected with a plasmid expressing shRNA specific for integrin αV: clones 2αV and 3αV. The surface expression of integrin subunit αV and integrin heterodimers αVβ3 and αVβ5 in both clones was substantially decreased, as measured by flow cytometry. Additionally, we observed slightly lower expression of these integrins in 3αV compared to 2αV. Since the MDA-MB-435S cell line does not express integrin αVβ1 (Taherian et al., 2011), the knockdown of integrin αV did not affect the expression of integrin β1 heterodimers (Figure 1). Both clones showed increased sensitivity to PTX and VCR and decreased sensitivity to cDDP (Figure 2A), which is in line with our previous results using transient transfection of integrin αV- or β5-specific siRNA (Stojanovic et al., 2018). In addition, 72 h after PTX treatment a greater number of apoptotic cells was found in the cultures of both clones 2αV and 3αV as compared to parental MDA-MB-435S cells (30 and 32% compared to 17%, respectively) (Figure 2B). Decreased expression of integrins αVβ3 and αVβ5 in clones 2αV and 3αV did not affect cell proliferation (Figure 2C). However, when cells were cultivated for 72 h, starting with the same number of plated cells, we observed decreased number of 2αV cells and particularly 3αV cells, showing a dose response relationship with integrin αV knockdown (Figure 2D). In addition, when culturing cells for 48 h to perform IF staining (Figure 3), we observed that clones 2αV and 3αV were smaller than MDA-MB-435S cells. We assumed that delayed adhesion to ECM proteins, due to integrin αV knockdown, was the main reason for this effect. In order to test this hypothesis, we visualized cell attachment and spreading using live cell microscopy for 12 h following plating (Figure 2E), monitored cell spreading within 72 h of growth (Supplementary Figure S1A), and analyzed cell surface area (Supplementary Figure S1B), size and granularity of cells in suspension using flow cytometry (Supplementary Figure S1C). Results showed that although all cells have similar initial rounded sizes in suspension, clones 2αV and 3αV have significantly impaired attachment and spreading when plated on culture dishes compared to parental cells. Finally, decreased migration of both clones compared to MDA-MB-435S cells was observed (Figure 2F), which is also in line with our previous results following transient transfection with integrin αV-specific siRNA (Stojanovic et al., 2018). The degree of inhibition of migration correlated with the extent of integrin αV knockdown (Figures 1, 2F).

Our recently published data have shown that upon transient knockdown of integrin subunit αV in MDA-MB-435S, the cells showed disorganization of actin with loss of stress fibers and significant loss of paxillin staining at the tips of stress fibers, indicating a reduction in the number of FAs (Stojanovic et al., 2018). We therefore tested whether clones 2αV and 3αV show similar features. IF staining of MDA-MB-435S cells confirmed the formation of well-defined adhesion structures containing integrin αV and paxillin while in clones 2αV and 3αV their number was reduced (Figure 3A). Furthermore, both clones had a decreased number of FAs per cell analyzed by counting either integrin αV- or paxillin-positive puncta, as well as a decreased amount of actin stress fibers per cell (Figure 3B). In conclusion, the model of MDA-MB-435S-derived stably transfected clones 2αV and 3αV fully mimics the effects of transient transfection with integrin αV-specific siRNA.

### Melanoma Cell Line MDA-MB-435S Primarily Utilizes Integrin αVβ5 for Adhesion

To better understand the observed changes in cell migration and sensitivity to microtubule poisons, PTX and VCR, the adhesome of MDA-MB-435S and clones 2αV and 3αV was analyzed. Since cytotoxicity assays (Figures 2A,B), as well as IF analysis (Figure 3), were performed in cell culture without prior coating with ECM proteins, we analyzed IACs in the same manner. This approach also enabled cell-secreted ECM proteins to be analyzed. We performed MS analysis of isolated IACs from MDA-MB-435S cells and clones 2αV and 3αV following 48 h of growth. IACs were isolated as previously described (Jones et al., 2015). The duration of crosslinking (see section “Materials and Methods”) was chosen based on the WB analysis of isolated IACs using antibodies against well-defined adhesion complex components, such as paxillin and non-receptor tyrosine kinase Src [pSrc (Y418)] (data not shown). IACs were then isolated from MDA-MB-435S and clones 2αV and 3αV and the isolation procedure performed in triplicate for MDA-MB-435S and 3αV and in duplicate for 2αV. Samples were analyzed by LC-MS/MS and spectral counts used as a measure of protein abundance. These analyses detected 153 proteins with at least 99% confidence from IACs isolated from MDA-MB-435S cells (Supplementary Table S2.1), including 120 proteins that were termed as meta-adhesome proteins (Horton et al., 2015). Label-free quantification demonstrated good reproducibility between data either from technical or biological replicates.

**FIGURE 4:**
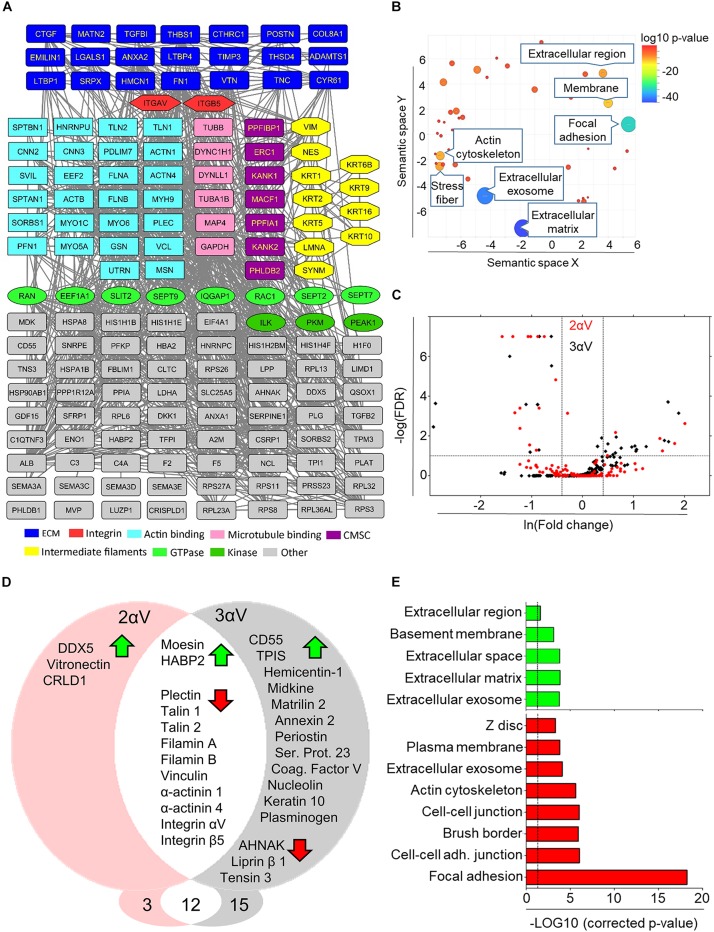
Mass spectrometry analysis of IACs isolated from MDA-MB-435S cells and clones 2αV and 3αV. **(A)** Protein–protein interaction network of components identified by MS in IACs isolated form MDA-MB-435S cells. Shapes represent identified proteins and are labeled with gene symbols, arranged and colored according to their functional group as indicated (CMSC). In case of multiple functional terms assigned for each protein, the molecular function assigned by both databases has been chosen for interpretation of results. **(B)** Total identified IAC proteins in MDA-MB-435S cells (number of spectral counts ≥ 4, FDR < 5%, probability for protein identification ≥ 99.9%) were annotated using David GO database. To determine the sample enrichment, *P*-values related to GO terms of cellular components (GOTERM_CC_DIRECT), were used. Analysis of gene ontology terms was performed using REViGO tool. Statistically significant GO terms (*P* = 0.05) were presented from highest *P*-value (bottom) to the lowest (top). **(C)** Volcano plot of MDA-MB-435S versus 2αV (red) and 3αV (black). To determine the significantly changed proteins −Log (FDR) ≥ 1 and fold change ≥ 1.5 were used. Upper left quadrant – proteins detected at lower level of spectra, upper right quadrant – proteins detected at higher level of spectra. **(D)** Venn diagram – proteins with higher (green arrow) and lower (red arrow) level of spectra detected in clones 2αV and 3αV versus MDA-MB-435S cells. Proteins found in both clones with changed abundances were showed in the intersected white area of the diagram. **(E)** DAVID GO analysis of proteins detected with higher (green arrow) and lower (red arrow) abundances. Statistically significant GO terms (*P* = 0.05; dashed line) were presented from highest *P*-value (bottom) to the lowest (top).

To provide an overall view of both components of the isolated IACs in MDA-MB-435S cells and of components of the ECM, a protein–protein interaction network was constructed (Figure 4A and Supplementary Table S2.2). The most surprising finding was that the only integrin receptor subunits identified in MDA-MB-435S cells were αV and β5, indicating that these cells primarily utilize integrin αVβ5 for adhesion in long term 2D culture (48 h). The majority of identified proteins were actin-binding and ECM proteins. We also identified proteins reported to bind MT and intermediate filaments, several GTPases and kinases, together with components of the CMSC (Noordstra and Akhmanova, 2017; Chen et al., 2018).

To further analyze the dataset, Gene Ontology (GO) enrichment analysis was performed on proteins identified in IACs isolated from MDA-MB-435S cells, using the online bioinformatics tools available via the Database for Annotation, Visualization and Integrated Discovery (DAVID^1^) (Huang da et al., 2009b), and GO terms were visualized using REViGO tool (Supek et al., 2011). Analysis confirmed a significant enrichment of GO terms related to ECM, extracellular exosome, FA and actin cytoskeleton (Figure 4B and Supplementary Table S2.3).

### Integrin αVβ5-Induced Interaction Networks

A volcano plot was constructed displaying proteins whose abundance was different in clones 2αV (red dots) or 3αV (black dots) compared to parental cells (Figure 4C). The differences observed in clones 2αV and 3αV, compared to MDA-MB-435S cells, were summarized using a Venn diagram (Figure 4D). As expected, both clones showed reduced levels of integrins αV and β5 compared to control cells. IAC proteins detected at lower levels in clones 2αV and 3αV are in the category of proteins detected by high number of spectra (Supplementary Table S2), and they belong mostly to the actin binding subgroup of the adhesome: plectin, talin 1 and 2, filamin A and B, α-actinin 1 and 4, and vinculin. Additionally, lower levels of three adhesome proteins, liprin β1, AHNAK and tensin 3, were found only in clone 3αV. We also observed higher levels of 17 proteins upon integrin αV knockdown in either 2αV (5), 3αV (14), or both (2) (Figure 4D), all belonging to the group of proteins identified by lower number of spectra (Supplementary Table S2.4). DAVID GO analysis of IAC proteins with lower abundance in clones 2αV or 3αV, compared to MDA-MB-435S cells, suggested that they are mostly components of FAs. Conversely, proteins present in higher levels in clones 2αV or 3αV as compared to MDA-MB-435S cells were classified as ECM or extracellular exosome proteins (Figure 4E and Supplementary Table S2.4).

We compared our adhesome protein dataset with those previously reported in the literature (Winograd-Katz et al., 2014; Horton et al., 2015). However, it should be noted that this adhesome dataset differed in that it was not obtained from cells seeded on fibronectin, but upon cultivation of cells for 48 h without prior coating with any ECM component. Therefore, it was not surprising that, unlike other reported adhesomes, we detected many ECM proteins secreted by the cells themselves. More specifically, from 153 proteins that we identified in our adhesome, 120 were reported in earlier studies (Supplementary Table S2.1). DAVID GO analysis of the remaining 33 proteins showed that they were mostly related with extracellular proteins (exosome and ECM proteins; Supplementary Table S2.5). In addition, we investigated whether any proteins were differentially expressed in our cell model which might indicate that they are specific for either the integrin αV adhesome or matrisome. We identified only one, i.e., Hyaluronan Binding Protein 2 (HABP2) for which the increased abundance had been observed in cell clones with decreased expression of integrin αV. Interestingly, we have observed the increased abundance of HABP2 in another melanoma cell line RPMI-7951 cell model upon transient knockdown of integrin αV (data not shown, manuscript in preparation). HABP2 is the extracellular serine protease which play a role in non-small lung cancer progression (Mirzapoiazova et al., 2015) but its role in melanoma is unknown.

**FIGURE 5:**
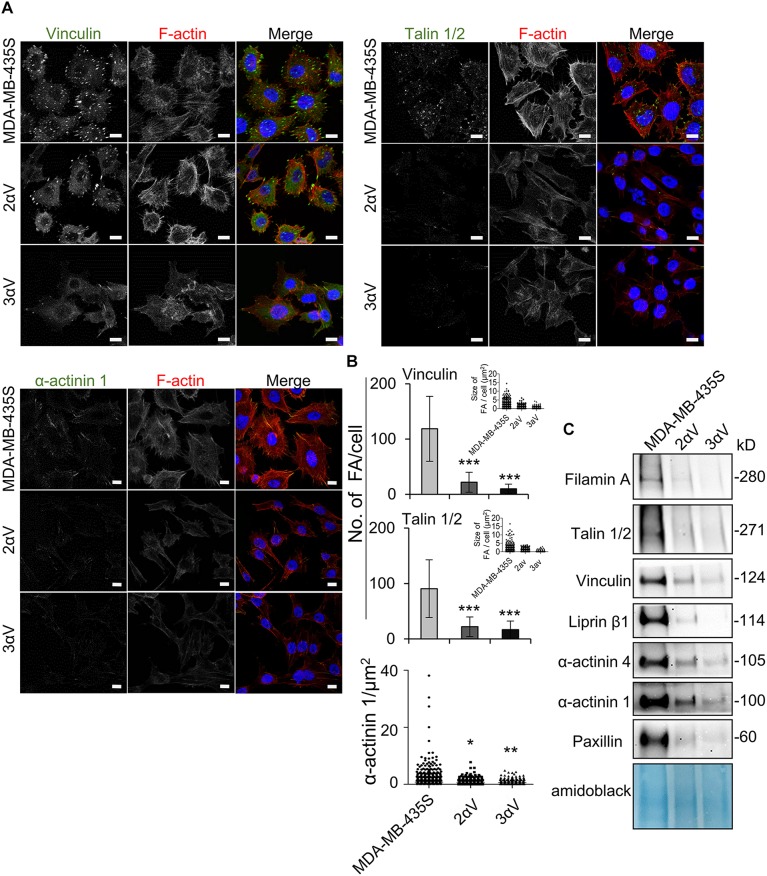
MS data validation in MDA-MB-435S cells and clones 2αV and 3αV. **(A)** Clones 2αV and 3αV show decreased expression of vinculin, talin 1/2 and α-actinin 1 as compared to MDA-MB-435S cells. Forty-eight hours after seeding on coverslips, cells were fixed, permeabilized, incubated with antibodies against vinculin, talin 1/2 or α-actinin 1 antibody, followed by Alexa-Fluor 488-conjugated antibody (green). F-actin staining (red) was performed in all samples, and nuclei were stained with DAPI (blue). Analysis was performed using TCS SP8 Leica. Scale bar = 10 μm. **(B)** Quantification data of results in **(A)** presented as histograms (FA number) and scatter plots (FA or α-actinin 1 size) represent measurements of > 50 cells and are plotted as mean ± SD (*n* = 3). Data were analyzed by one-way ANOVA with Dunnett’s multiple comparison. *P < 0.05; **P < 0.01; ***P < 0.001. **(C)** WB analysis of IAC proteins from clones 2αV and 3αV and MDA-MB-435S cells. Forty-eight hours after seeding, IACs were isolated and WB analysis was performed. The results presented are representative of three independent experiments yielding similar results.

Next we validated data from MS using IF and WB. We selected two proteins in clone 2αV with the largest decrease, vinculin and talin, and one protein with a low but still significant decrease, α-actinin 1 (Supplementary Table S2). IF analysis showed a lower number of FAs per cell according to vinculin or talin 1/2 labeling, as well as a reduced area of α-actinin 1 labeling in both clones compared to MDA-MB-435S cells (Figures 5A,B). Of note, although they were not identified by MS, we analyzed the expression of pFAK (Y397) and pSrc (Y418) using IF and found changes in their expression, which is in line with the decreased number of FAs (Supplementary Figure S2). Finally, WB was performed on IAC isolates, which confirmed decreased levels of filamin A, talin 1/2, vinculin, α-actinin 1, α-actinin 4, liprin β1, and FA marker protein paxillin (Figure 5C and Supplementary Figure S5).

### Integrin αVβ5 Adhesion Complexes Are Composed of Focal and Reticular Adhesions

**FIGURE 6:**
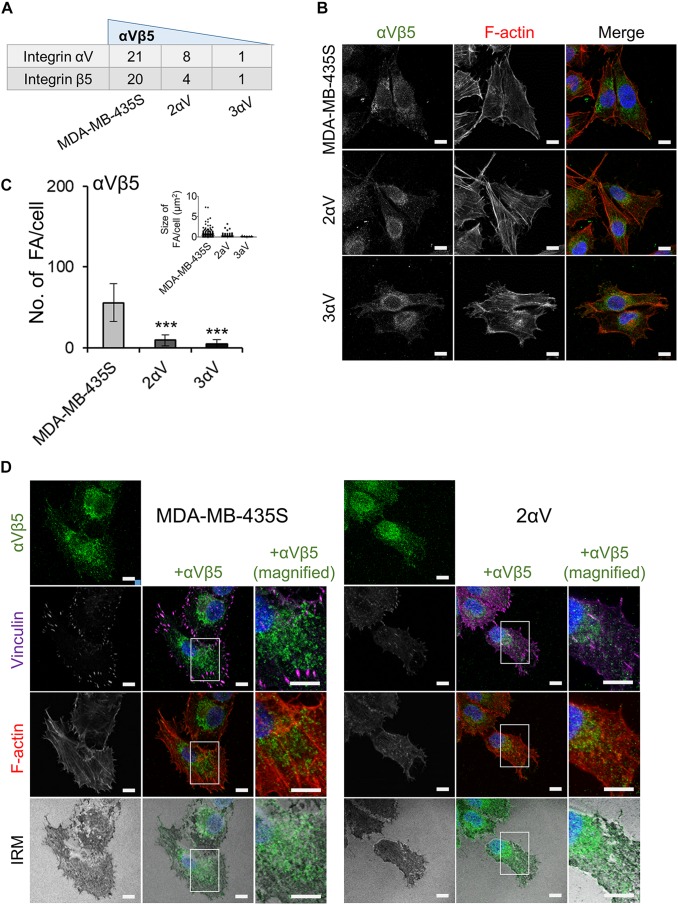
Characterization of αVβ5-associated adhesion complexes. **(A)** The most abundant integrin subunits found in IACs. Dataset consists of at least two different experiments. Average spectral count number shown. **(B)** Clones with integrin subunit αV knockdown show decreased expression of integrin αVβ5. Forty-eight hours after seeding on coverslips, cells were fixed, permeabilized, and stained with anti-αVβ5 antibody, followed by Alexa-Fluor 488-conjugated antibody (green). F-actin staining (red) was performed, and nuclei were stained with DAPI (blue). Analysis was performed using TCS SP8 Leica. Scale bar = 10 μm. **(C)** Quantification data of results in **(B)** presented as histogram (FA number) and scatter plot (FA size) represent measurements of > 50 cells and are plotted as mean ± SD (*n* = 2). Data were analyzed by one-way ANOVA with Dunnett’s multiple comparison. ***P < 0.001. **(D)** Identification of reticular adhesion structures in MDA-MB-435S cells and clone 2αV. Forty-eight hours after seeding on coverslips, cells were fixed, permeabilized, and stained for anti-αVβ5 followed by Alexa-Fluor 488-conjugated antibody (green), and anti-vinculin followed by Alexa-Fluor 647-conjugated antibody (purple). F-actin staining (red) was performed, nuclei were stained with DAPI (blue) and IRM images were taken. Analysis was performed using TCS SP8 Leica. Scale bar = 10 μm.

Our results have shown that integrin αVβ5 is the preferential integrin used in cell culture by melanoma cell line MDA-MB-435S (Figure 4). Stable knockdown of integrin αV in clones 2αV and 3αV decreased the number of integrin αV and β5 peptides detected by MS (Figure 6A), which correlated with the results of measurement of integrin αVβ5 surface expression by flow cytometry (Figure 1). We have also quantified the number of FAs per cell according to integrin αVβ5 heterodimer-specific antibody staining (Figures 6B,C) and observed a similar dose-response relationship as already observed for integrin αV and paxillin (Figures 3A,B) or talin 1/2 or vinculin (Figures 5A,B). Interestingly, IF analysis has shown that integrin αVβ5 in MDA-MB-435S was also localized further from the cell periphery, forming ring-like or reticular structures, but without recruiting vinculin (Figure 6D, left panel). These structures resembled recently described RAs shown to maintain cell–ECM attachment during mitotic rounding and division (Lock et al., 2018). The FAs and RAs were shown to colocalize with dark areas in IRM, indicating their presence at the cell-substrate adhesion sites. Reticular adhesions were also present in cell clone 2αV and also colocalized with dark IRM areas (Figure 6D, right panel). Lock et al. (2018) observed a range of defects in β5 depleted cells, including the failure of cytokinesis which resulted in binucleate daughter cells. Indeed, in MDA-MB-435S cells upon integrin αV knockdown we observed more multinucleated cells (Supplementary Figure S3).

### KANK2 Is a Potential Key Target for Increasing Sensitivity to Microtubule Poisons and Decreasing Migration

A recent report (Bouchet et al., 2016) demonstrated the existence of CMSC in the vicinity of mature FAs that capture MTs, containing CLASPs, kinesin family member 21A (KIF21A), LL5β [also known as pleckstrin homology (PH)–like domain, family B, member 2 (PHLDB2)], liprin α1 and β1, as well as paralogs of KANK. They showed that the CMSC is recruited to FAs by KANK1, which directly interacts with the major FA component, talin. On the other hand, KANK2 was also found adjacent to FAs in regions enriched in liprin β1 and ELKS (known as ERC1 for ELKS/RAB6-interacting/CAST family member 1), and was shown to bind talin directly to MTs (Sun et al., 2016).

**FIGURE 7:**
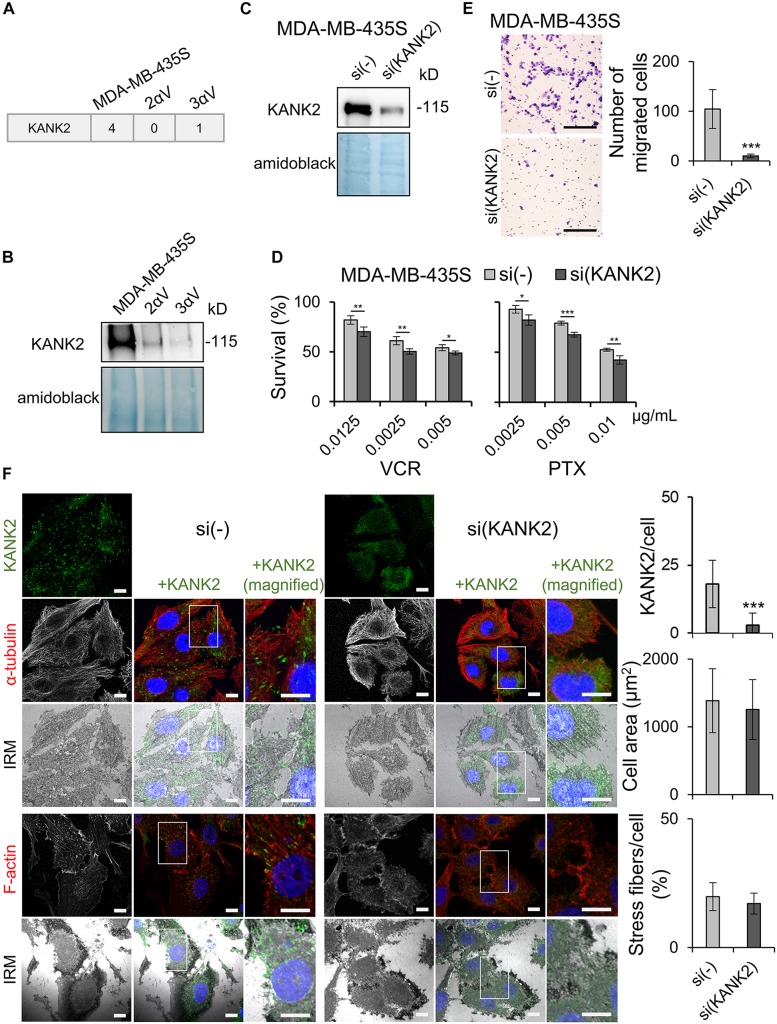
KANK2 knockdown in MDA-MB-435S cells increases sensitivity to MT poisons and decreases migration mimicking integrin αV knockdown. **(A)** Average MS data of number of spectra specific for KANK2 in MDA-MB-435S cell model. Dataset consists of at least two different experiments. **(B)** KANK2 is present in lower amount in IACs of clones 2αV and 3αV. WB analysis of KANK2 in isolated IAC proteins from clones 2αV and 3αV and MDA-MB-435S cells. Forty-eight hours after seeding, IACs were isolated and WB analysis was performed. The results presented are representative of two independent experiments yielding similar results. **(C)** Knockdown of KANK2 in MDA-MB-435S cells. WB analysis of KANK2 from MDA-MB-435S cells transfected with either control (si(-)) or KANK2-specific siRNA (si(KANK2)). Forty-eight hours after transfection total cell lysates were collected and WB analysis was performed. The results presented are representative of two independent experiments yielding similar results. **(D)** MDA-MB-435S cells upon KANK2 knockdown demonstrate increased sensitivity to VCR and PTX as compared to MDA-MB-435S cells transfected with control siRNA. Twenty-four hours upon transfection, cells were seeded in 96-well plates and 24 h later treated with different concentrations of VCR and PTX. Cytotoxicity was measured by MTT assay. Results presented are representative of three independent experiments with similar results ± SD. Data were analyzed by unpaired Student’s *t*-test. *P < 0.05; **P < 0.01; ***P < 0.001. **(E)** KANK2 knockdown decreases migration in MDA-MB-435S cells. Serum starved (24 h) cells, transfected previously with either control or KANK2-specific siRNA were seeded in Transwell cell culture inserts and left to migrate for 22 h toward serum. Cells on the underside of the inserts were stained with crystal violet, photographed, and counted. Scale bar = 100 μm. Averages of five microscope fields of three independently performed experiments ± SD are shown (*n* = 3). Data were analyzed by one-way ANOVA with Dunnett’s multiple Comparison. ***P < 0.001. **(F)** KANK2 knockdown in MDA-MB-435S cells does not alter cell size or amount of stress fibers but slightly alters appearance of MTs. Forty-eight hours after transfection of MDA-MB-435S cells with KANK2-specific or control siRNA cells were fixed, permeabilized, and stained with anti-KANK2 antibody, followed by Alexa-Fluor 488-conjugated antibody (green). The α-tubulin or F-actin staining (red) was performed, nuclei were stained with DAPI (blue) and IRM images were taken. Analysis was performed using TCS SP8 Leica. Scale bar = 10 μm. Quantification data of results are presented as histograms represent measurements of > 50 cells and are plotted as mean ± SD (*n* = 2). Data were analyzed by one-way ANOVA with Dunnett’s multiple comparison. ***P < 0.001.

As described above, in one of the clones (3αV), we observed a reduced abundance of liprin β1 in IACs (Figure 4D), which is a key component of CMSC (Bouchet et al., 2016). However, assessment of MS data (Supplementary Table S2.1, marked as YES in CMSC column) showed that liprin β1, although not statistically significant, was also present at a lower abundance in cell clone 2αV. The reduced abundance of liprin β1 was confirmed in both clones using WB analysis (Figure 5C). Further inspection of the MS data identified several other CMSC proteins in MDA-MB-435S and clones 2αV and 3αV, although represented at a lower abundance. These were liprin α1, ELKS, LL5β, MT-actin cross-linking factor 1 (MACF1), and KANK1 and 2 (Supplementary Table S2.1, marked as YES in CMSC column). Since KANK proteins recently emerged as key regulators of adhesion dynamics (Chen et al., 2018), we selected KANK2 for further investigation. We observed lower number of average spectra for KANK2 in clones 2αV and 3αV compared to MDA-MB-435S cells (Figure 7A), and confirmed lower expression by WB in samples of isolated IACs (Figure 7B and Supplementary Figure S6).

In order to analyze the role of KANK2 in sensitivity to MT poisons, we measured sensitivity to PTX and VCR upon transient transfection of MDA-MB-435S cells with control vs. KANK2-specific siRNA. Knockdown of KANK2 in MDA-MB-435S cells was successful (Figure 7C and Supplementary Figure S6), and resulted in increased sensitivity to VCR and PTX (Figure 7D). Finally, we demonstrated that migration of MDA-MB-435S cells transfected transiently with KANK2-specific siRNA was reduced in comparison to cells transfected with control siRNA (Figure 7E). We conclude that KANK2 is a potential target IAC protein for increasing sensitivity to MT poisons and decreasing migration.

Crosstalk between actin and MT dynamics is mediated through a number of proteins (Garcin and Straube, 2019). Among them is the KANK family of proteins, which play important roles in regulating MT dynamics around FAs (Bouchet et al., 2016; Chen et al., 2018). KANK2, MTs and the actin cytoskeleton were therefore visualized in MDA-MB-435S cells with and without KANK2 knockdown. Simultaneously, IRM was used to visualize FAs. In control cells, we found KANK2 at the tips of MT fibers that overlapped with FAs. Although the cell size did not change upon KANK2 knockdown, the MTs did appear more condensed. Visualization of actin upon KANK2 knockdown showed that the amount of stress fibers did not change (Figure 7F).

Finally, to test whether KANK2 is predominantly linked to integrin αVβ5, we analyzed KANK2 localization upon integrin β5 knockdown (Supplementary Figure S4A). Results showed strongly reduced levels of KANK2-positive puncta, reduced amount of stress fibers and reduced cell size (Supplementary Figures S4B,C). We conclude that KANK2 is a key molecule linking integrin αVβ5 FAs to MTs, thus enabling actin-MT crosstalk that is important for both sensitivity to MT poisons and cell migration.

## Discussion

αV integrins, which have been implicated in tumor growth and angiogenesis, as well as in sensitivity to chemo- and radiotherapy, have long been recognized as therapeutic targets. However, there are several factors that complicate the development of integrin-based therapeutics for cancer, and deepening the basic knowledge about integrin αV-adhesion complexes is thus necessary. Recent advances in proteomics have enabled researchers to study IAC composition (adhesome) in detail (Humphries et al., 2019). Here, we assessed αV-dependent changes in the adhesome of MDA-MB-435S cells to understand better the increased sensitivity to PTX and VCR and decreased migration observed in this cell line upon integrin αV knockdown. Our major finding is that KANK2 provides a link between IACs and MTs that determines sensitivity to MT poisons.

Analysis of the MDA-MB-435S adhesome detected only αV and β5 integrin subunits, thus showing that in long term culture these cells use preferentially integrin αVβ5 for adhesion. This result is in accordance with recently published results by Lock et al. (2018), who found predominantly αV and β5 subunits in the adhesome of human osteosarcoma U2OS, lung carcinoma A549 and melanoma A375 cells in long term culture, whereas other integrin α and β subunits were present at much lower levels. Although we did not detect other integrin subunits, we do not exclude the possibility that MDA-MB-435S cells use integrin αVβ3 or small amounts of other integrin heterodimers in adhesion.

Among the ECM proteins detected, vitronectin (Takada et al., 2007) and periostin (Gillan et al., 2002) are able to bind integrin αVβ5. Major group of cytoskeletal proteins detected in the adhesome were actin-binding proteins, together with several MT-binding and intermediate filament proteins. However, the striking characteristic of MDA-MB-435S adhesome was detection of several proteins from the CMSC (liprin α1 and β1, ELKS, LL5β, MACF1, KANK1 and 2), the punctate structures behind the lamellipodium that cluster in the vicinity of mature FAs (Bouchet et al., 2016; Chen et al., 2018).

Statistical comparison of adhesomes from clones 2αV and 3αV to MDA-MB-435S adhesome showed reduced abundance of liprin β1 in clone 3αV. In addition, WB analysis of isolated IAC proteins confirmed reduced abundance of liprin β1 in both 2αV and 3αV clones, compared to MDA-MB-345S cells. In melanoma, liprin β1 was found to be overexpressed and its expression strongly correlated with the expression of KANK1 and KANK2 (Luo et al., 2016). Talin 1 and liprin β1, both showing reduced abundance in clones 2αV and 3αV, were identified as binding partners of KANK2 (Sun et al., 2016; Weng et al., 2018). Inspection of MS data revealed that, despite a lower number of spectra for CMSC proteins in our cell model, clones 2αV and 3αV show slightly reduced abundance compared to MDA-MB-435S cells, especially for KANK2. By combining these results with literature data on the role of KANK2 as a linker between talin and MTs (Bouchet et al., 2016; Chen et al., 2018), we hypothesized that KANK2 is the key protein involved in increased sensitivity to MT poisons and decreased cell migration. Indeed, KANK2 knockdown in MDA-MB-435S cells mimics increased sensitivity to MT poisons and decreased migration previously shown in αV knockdowns. Our MS data demonstrated small but similar number of spectra for KANK1 and KANK2, thus indicating their similar expression. Therefore, reduced expression of KANK1 as well as similar role of KANK1 in sensitivity to MT poisons cannot be excluded. It is well-known that three out of four KANK family members contribute to microtubule targeting (Chen et al., 2018) and addressing the issue of isoform-specific roles will be the subject of a further study.

IF analysis did not show striking changes in actin and MT cytoskeleton upon KANK2 knockdown in MDA-MB-435S cells, unlike integrin αV knockdown, which led to disorganization of actin with loss of stress fibers. This indicates that KANK2 knockdown in MDA-MB-435S cells did not disrupt FAs nor significantly changed the appearance of the MT cytoskeleton. This conclusion is supported by unchanged cell spreading area upon KANK2 knockdown, whereas knockdown of integrin αV decreased the cell spreading area. This is in line with depletion of KANK2 in fibroblasts or KANK1 in HeLa cells, which both did not result in any obvious alteration of FAs size or disassembly (Bouchet et al., 2016; Sun et al., 2016). We tested the effect of two antitumor drugs with the opposite mechanism of action, PTX which stabilizes and VCR which destabilize microtubules. The increased sensitivity of MDA-MB-435S cells to both MT poisons upon integrin αV or KANK2 knockdown points to altered MT dynamics in these cells. Consistently, recent data in other cell models have shown that KANK proteins mediate crosstalk between actin and MT cytoskeletons at FAs (Bouchet et al., 2016; Sun et al., 2016; Chen et al., 2018; Rafiq et al., 2019).

The question that arises is whether integrin αVβ5 FAs in MDA-MB-435S cells are the only ones linked to CMSCs through KANK2. Since the KANK localization to adhesions is primarily through talin (Gough and Goult, 2018) it is very likely that integrin αVβ3 FAs contain KANK2 as well. Our previously published results have shown that knockdown of either integrin β5 or β3 did not change cell migration, while knockdown using integrin αV-specific siRNA of both heterodimers αVβ3 and αVβ5, the only integrins containing αV expressed in MDA-MB-435S cells, dramatically inhibited migration. Additionally, integrin β5 knockdown increased sensitivity of MDA-MB-435S cells to MT poisons, but not as efficiently as integrin αV knockdown, while integrin β3 knockdown had an opposite effect. We explained these results by a balance effect in MDA-MB-435S cell line in which integrin β5 knockdown increased the expression of integrin αVβ3 heterodimer on the cell surface and vice versa, and concluded that integrin αVβ5 is the one responsible for sensitivity to MT poisons while both integrins αV (αVβ3 and αVβ5) are implicated in cell migration (Stojanovic et al., 2018). KANK2 knockdown would knockdown microtubule localization to all integrin adhesions, whereas β5 knockdown would knock it down only to β5 integrins, so it is not surprising that the effect on cell migration is more pronounced. In conclusion, our results indicate that targeting KANK2 might simultaneously increase sensitivity to MT poisons therapy and decrease migration. Cell-specific effects of KANK 1 or 2 knockdown have been reported: in HeLa cells and podocytes KANK1 knockdown was shown to reduce motility (Li et al., 2011; Gee et al., 2015), while KANK2 knockdown in mice kidney fibroblasts increased migration (Sun et al., 2016).

In summary, the data presented here identify αVβ5 as the main integrin used by MDA-MB-435S cells to make both FAs and RAs. MS analysis revealed many proteins associated with integrin αVβ5 and represents the first published adhesome of this integrin heterodimer. KANK2 was shown to be associated with integrin αVβ5 since its knockdown mimicked initially observed integrin αV knockdown effects. Therefore, we propose KANK2 as a potential target for increasing sensitivity of melanoma cells to MT poisons and decreasing migration. These data will enable follow-up analyses of signaling mediated by integrin αVβ5 and therefore represent a valuable resource to improve our understanding of the mechanisms involved in the adhesion control of melanoma cell sensitivity to MT poisons and cell migration.

## Data Availability Statement

The mass spectrometry proteomics data have been deposited to the ProteomeXchange repository with the dataset identifier PXD016837. Other raw data supporting the conclusions of this article will be made available by the authors, without undue reservation, to any qualified researcher.

## Author Contributions

AA-R and MP contributed to the study conception and design. MP, JH, NS, DN, DM, AD, IS, DS, IW, MH, and AA-R performed research by material preparation, data collection and analysis. The first draft of the manuscript was written by AA-R while MP, MH, NS, and JH commented on previous versions of the manuscript. All authors read and approved the final manuscript.

## Conflict of Interest

The authors declare that the research was conducted in the absence of any commercial or financial relationships that could be construed as a potential conflict of interest.

## Footnotes

1 http://david.abcc.ncifcrf.gov/home.jsp

## Abbreviations

AHNAK: neuroblast differentiation-associated protein
ANOVA: related-measure two-way analysis of variance
cDDP: cisplatin
CLASP: cytoplasmic linker-associated proteins
CMSC: cortical microtubule stabilizing complex
DMEM: Dulbecco’s modified Eagle’s medium
DMSO: dimethyl sulfoxide
DTBP: dimethyl 3,3 ′ -dithiobispropionimidate Wang and Richard’s reagent
ECM: extracellular matrix
EDTA: ethylenediaminetetraacetic acid
EdU: 5-ethynyl-2 ′ -deoxyuridine
ELKS: ELKS/RAB6-interacting/CAST family member 1
FA: focal adhesion
FAK: focal adhesion kinase
FBS: fetal bovine serum
FN: fibronectin
HEPES: hydroxyethyl piperazineethanesulfonic acid
IAC: integrin adhesion complex
IF: immunofluorescence
ILK: integrin linked kinase
IRM: interference reflection microscopy
KANK: KN motif and ankyrin repeat domain-containing proteins
KIF21A: kinesin family member 21ALL5 β, pleckstrin homology (PH)–like domain family B member 2 (PHLDB2)
MACF1: MT-actin cross-linking factor 1
MS: mass spectrometry
MT: microtubule
MTT: 3-((4,5-dimethylthiazol-2-yl)-2,5-diphenyltetrazolium bromide)
PBS: phosphate-buffered saline
PINCH: particularly interesting new cys-his protein 1
PTX: paclitaxel
RA: reticular adhesion
Src: non-receptor tyrosin kinase Src
VASP: vasodilator-stimulated phosphoprotein
VCR: vincristine
WB: western blot WB.
