# Supplementary material for: KANK2 Links αVβ5 Focal Adhesions to Microtubules and Regulates Sensitivity to Microtubule Poisons and Cell Migration
Source: Front Cell Dev Biol. 2020 Mar 3;8:125. doi: 10.3389/fcell.2020.00125 (PMC7063070; doi:10.3389/fcell.2020.00125)
Supplement: Supplementary file 1 [file Data_Sheet_1.PDF]

Supplementary Material

Supplementary Figure 1

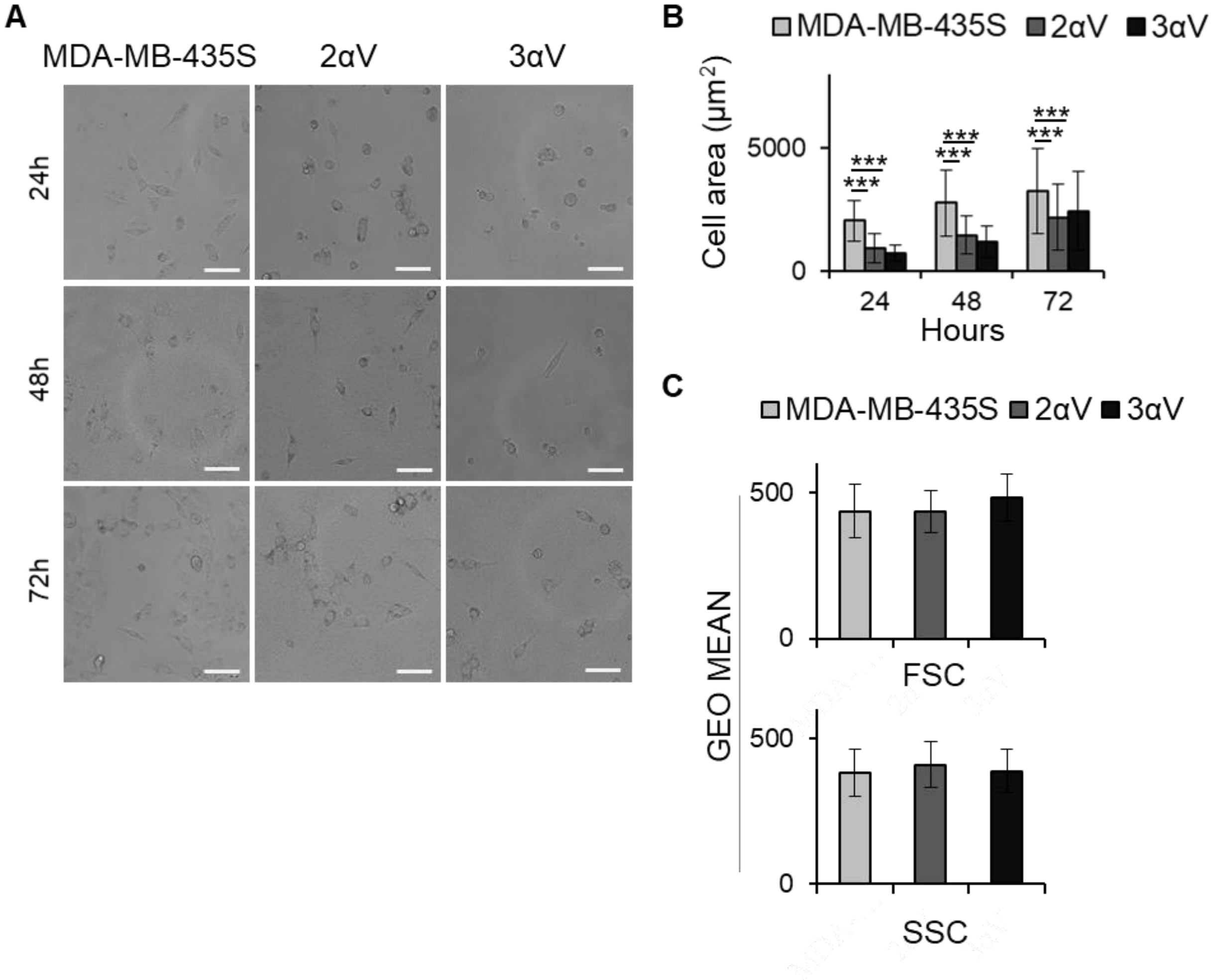

Supplementary Figure S1. Although all cells are equal in size, MDA-MB-435 cells spread faster than clones 2αV and 3αV, related to Figure 2 and 3. (A) Cell surface area comparison of MDA-MB-435S, 2αV and 3αV cells. Cells were seeded in 6-well dishes and imaged every 24 hours during a 72 h period using EVOS cell imaging system. Scale bar = 100 μm. **(B)** Cell size quantification data i.e. average of five microscope fields of three independently performed experiments ± S.D. shown as histogram. Data were analyzed by student t-test. ***P, 0.001. **(C)** Comparison of forward and side scatter using flow cytometry. The results presented are average values from at least five independent experiments.

Supplementary Figure 2

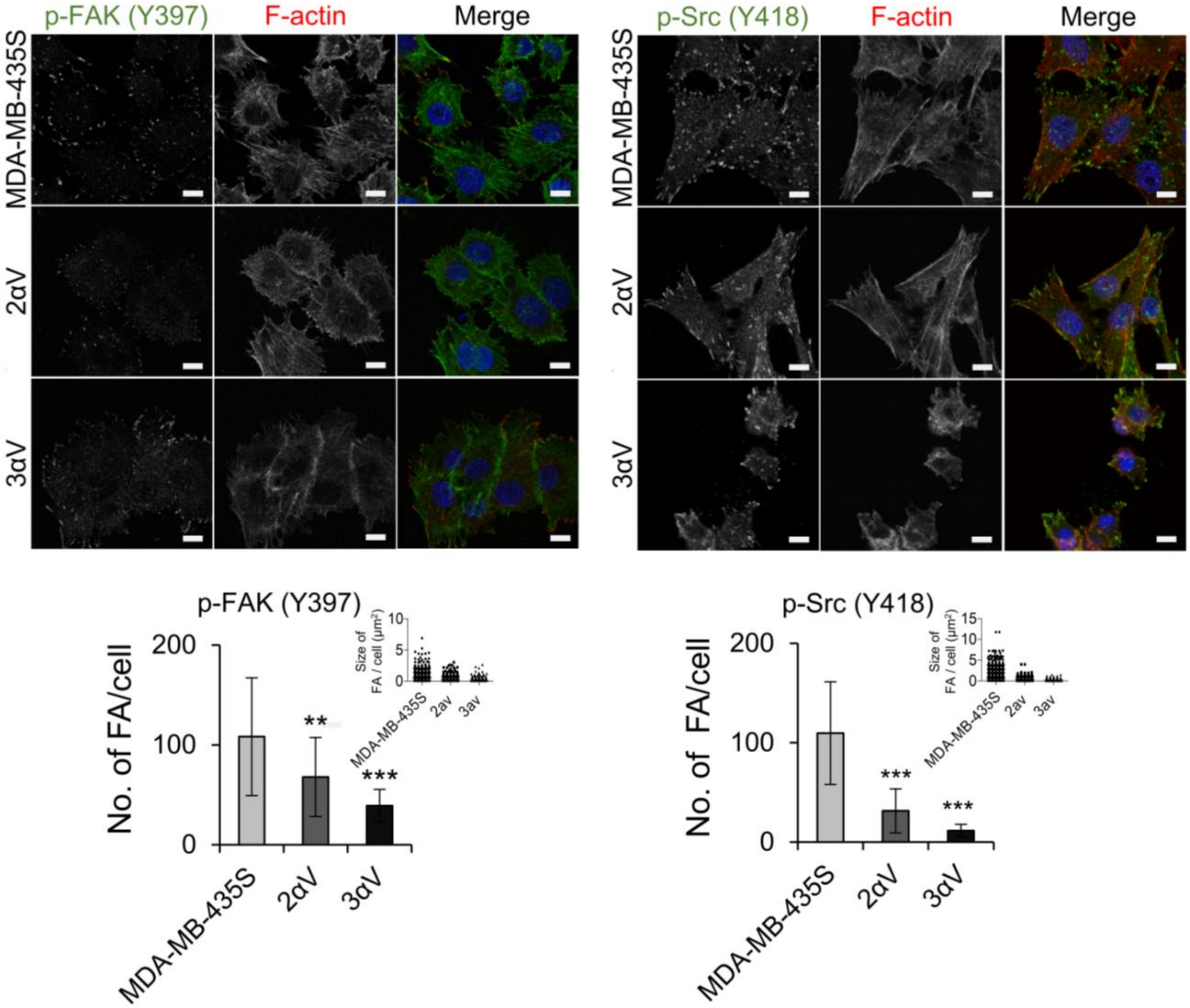

Supplementary Figure S2. Clones 2αV and 3αV show decreased expression of pFAK (Y397) and pSrc (Y418) as compared to the parental cell line. Forty-eight hours after seeding on coverslips, cells were fixed, permeabilized, and stained for anti-phospho-FAK (Y397) or anti-phospho-Src (Y418) antibody, followed by Alexa-Fluor 488-conjugated antibody (green). F-actin staining (red) was performed in all samples, and nuclei were stained with DAPI (blue). Analysis was performed using TCS SP Leica. Scale bar = 10 μm. Quantification data presented as histograms and scatter plots (presenting FA size) represent measurements of >50 cells and are plotted as mean ± S.D. (n = 3). Data were analyzed by one-way ANOVA with Dunnett's multiple comparison. **P, 0.01; ***P, 0.001.

Supplementary Figure 3

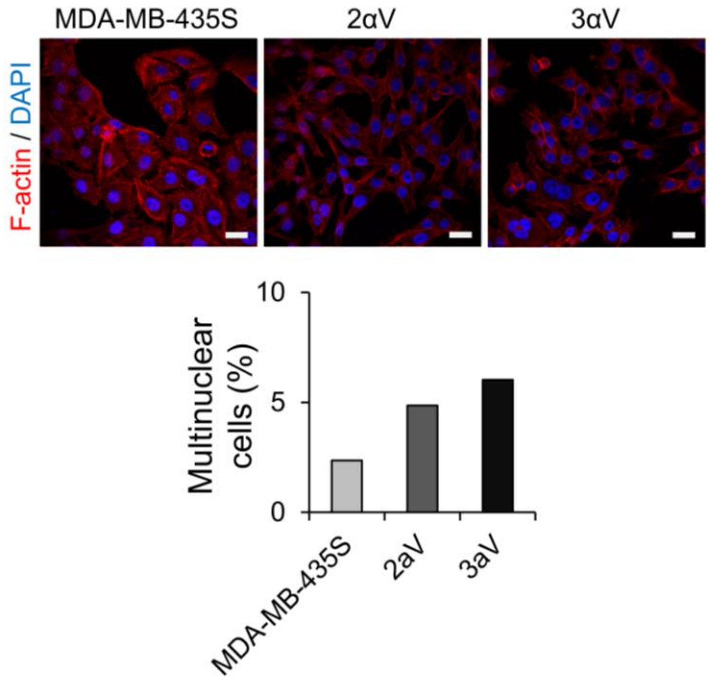

Supplementary Figure S3. Clones with integrin subunit αV knockdown have more multinuclear cells than the parental MDA-MB-435S cells. Forty-eight hours after seeding on coverslips, cells were fixed, permeabilized, and stained for F-actin (red). Nuclei were stained with DAPI (blue). Analysis was performed using TCS SP8 Leica. Scale bar = 25 µm. Quantification data presented as histograms represent measurements of >300 cells (n = 1).

Supplementary Figure 4

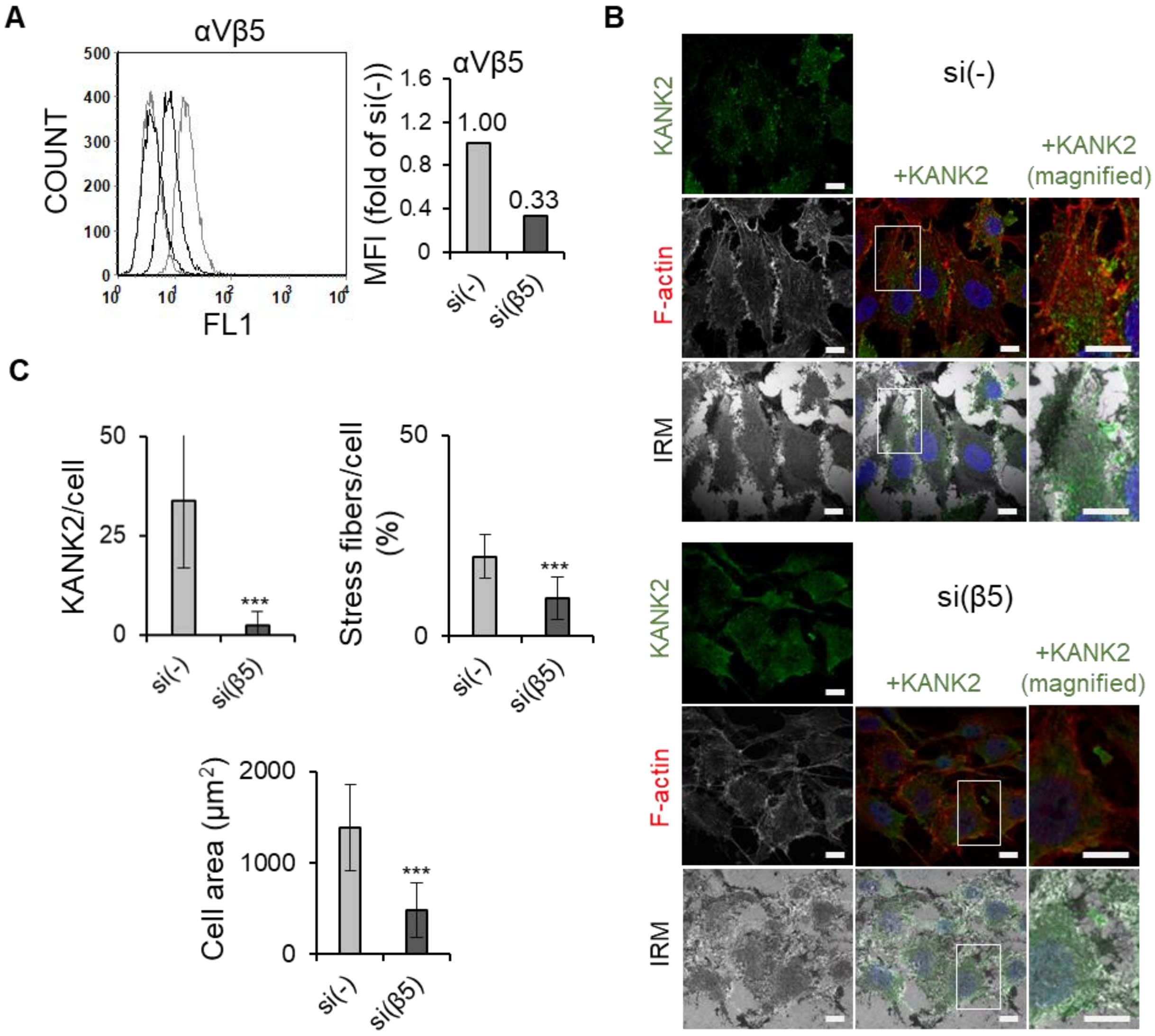

Supplementary Figure S4. Knockdown of β5 in MDA-MB-435S cells decreases expression of KANK2, the amount of stress fibres and reduces the cell size, related to Figure 7. (A) Surface expression of integrin αvβ5 in MDA-MB-435S cells was analyzed by indirect flow cytometry 48 hours after transfection with integrin subunit β5-specific siRNA (black histogram) and compared with cells transfected with control siRNA (gray histogram). Representative data of three independent experiments yielding similar results are shown, as well as Mean fluorescence intensities (MFI) relative to cells transfected with control siRNA for the corresponding graph. **(B)** Forty-eight hours upon silencing on cells seeded on coverslips, cells were fixed, permeabilized, and stained for anti-KANK2 antibody, followed by Alexa-Fluor 488-conjugated antibody (green). Actin staining (red) was performed in all samples, nuclei were stained with DAPI (blue) and IRM images were taken. Analysis was performed using TCS SP Leica. Scale bar = 10 µm. **(C)** Quantification data from **(B)** presented as histograms represent measurements of >50 cells and are plotted as mean ± S.D. (n = 2). Data were analyzed by one-way ANOVA with Dunnett's multiple comparison. ***P , 0.001.

Supplementary Figure 5

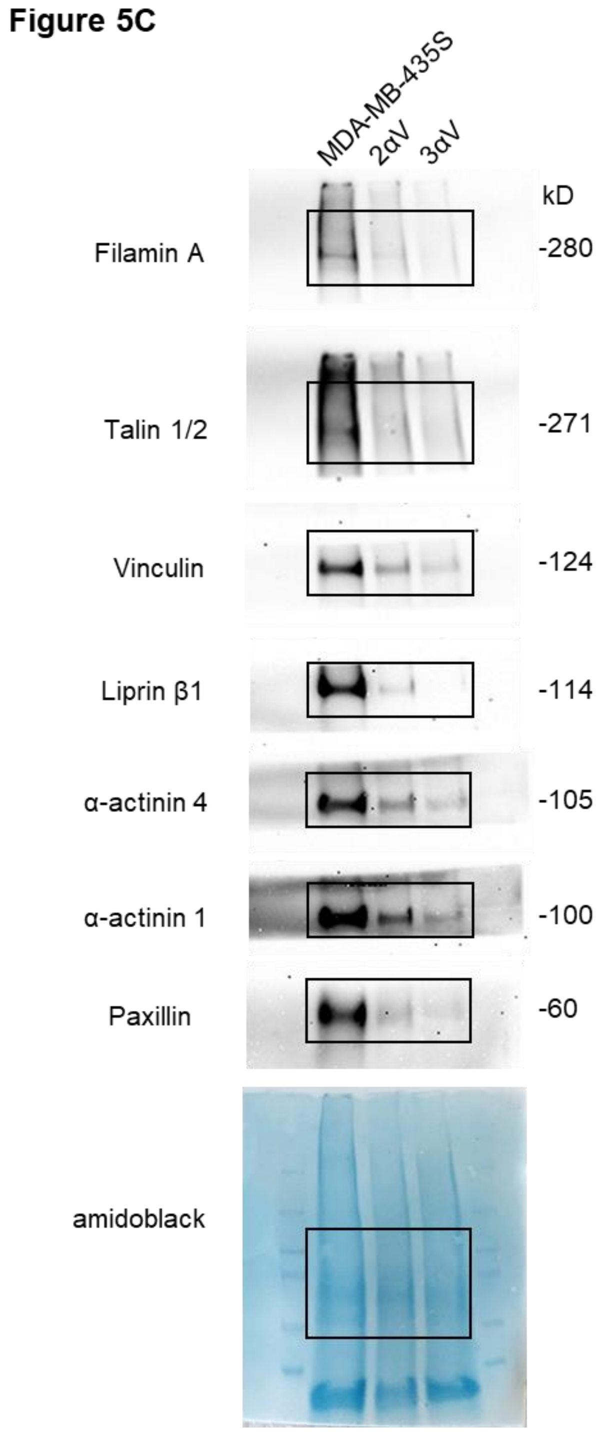

Supplementary Figure S5. Full images of the blots in Figure 5C. Images were obtained using iBright CL1000, which directly scanned membranes developed with ECL reagents.

Supplementary Figure 6

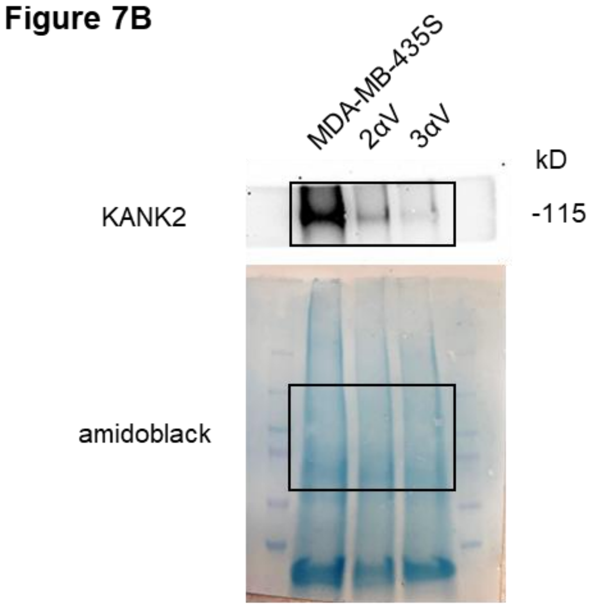

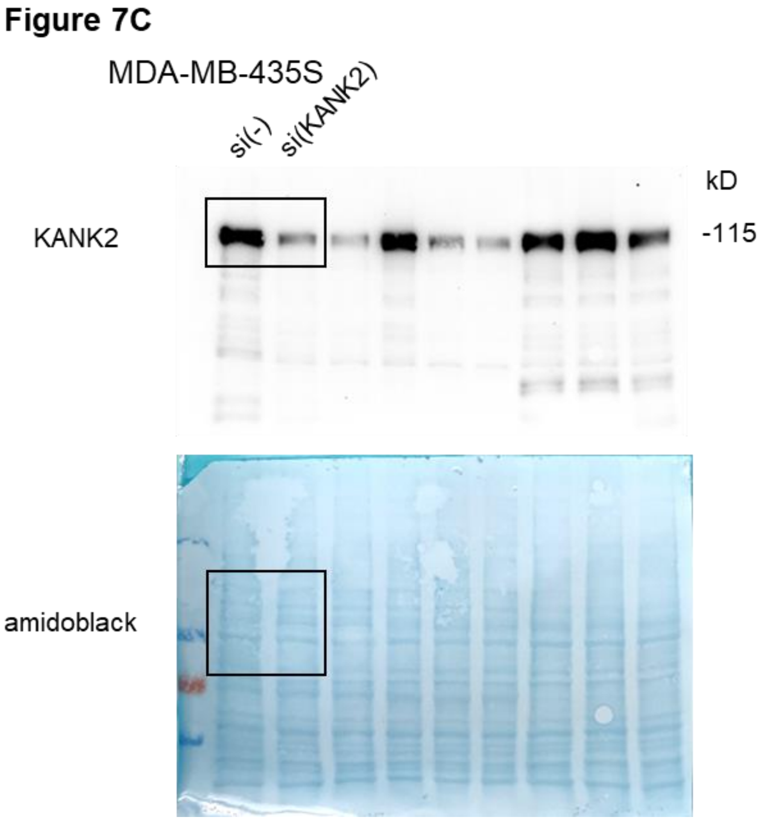

Supplementary Figure S6. Full images of the blots in Figure 7B and C. Images were obtained using iBright CL1000, which directly scanned membranes developed with ECL reagents.

Supplementary Table 1 List of antibodies and stains used in this study.

				Dilution		
**Primary antibodies**	**Cat. No.**	**Distributor**	**Species**	**FC**	**IF**	**WB**
IgG1 isotype control from murine myeloma	M5284	Sigma-Aldrich, USA	mouse	1:50		
Integrin αV (Ab-1), clone 272-17E6	407286	Merck Millipore, Germany	mouse	1:100		
Integrin αVβ3, clone LM609	MAB1976	Merck Millipore, Germany	mouse	1:100		
Integrin αVβ5, clone P1F6	MAB1961	Merck Millipore, Germany	mouse	1:100	1:50	
Integrin β1, clone JB1A	MAB1965	Merck Millipore, Germany	mouse	1:100		
Integrin αV, clone 272-17E6	MABT207	Merck Millipore, Germany	mouse		1:50	
FAK (phospho Y397), clone EP2160Y	ab81298	Abcam, USA	rabbit		1:100	
Src (phospho Y418)	ab4816	Abcam, USA	rabbit		1:100	
Filamin A, clone E-3	sc-17749	Santa Cruz, USA	mouse			1:500
Talin 1/ 2, clone 8D4	ab11188	Abcam, USA	mouse		1:25	1:1000
Vinculin, clone EPR8185	ab129002	Abcam, USA	rabbit		1:50	1:10000
Liprin β1, clone F-2	sc-514575	Santa Cruz, USA	mouse			1:250
α-actinin-4, clone G-4	sc-390205	Santa Cruz, USA	mouse			1:250
α-actinin-1, clone H-2	sc-17829	Santa Cruz, USA	mouse		1:50	1:500
Paxillin, clone Y113	ab32084	Abcam, USA	rabbit		1:100	1:7500
α-tubulin, clone B-7	sc-5286	Santa Cruz, USA	mouse		1:100	
KANK2	HPA015643	Merck Millipore, Germany	rabbit		1:200	
**Secondary antibodies**						
FITC Goat anti-Mouse Ig	554001	BD Pharmingen, USA	goat	1:50		
Donkey anti-Rabbit IgG (H+L), Alexa Fluor 488	A-21206	Thermo Fisher Scientific, USA	donkey		1:1000	
Anti-Rabbit IgG (H+L), Alexa Fluor 647	#4414	Cell Signaling Technology, USA			1:1000	
Goat anti-Mouse IgG1, Alexa Fluor 488	A-21121	Thermo Fisher Scientific, USA	goat		1:1000	

**Stain**						
Alexa Fluor 555 Phalloidin	#8953	Cell Signaling Technology, USA			1:40	
